# The Importance of Nature Exposure and Physical Activity for Psychological Health and Stress Perception: Evidence From the First Lockdown Period During the Coronavirus Pandemic 2020 in France and Germany

**DOI:** 10.3389/fpsyg.2021.623946

**Published:** 2021-03-04

**Authors:** Florian Javelle, Sylvain Laborde, Thomas Jean Hosang, Alan James Metcalfe, Philipp Zimmer

**Affiliations:** ^1^Clinical Exercise-Neuroimmunology Group, Department for Molecular and Cellular Sports Medicine, Institute for Cardiovascular Research and Sports Medicine, German Sport University, Cologne, Germany; ^2^Department of Performance Psychology, Institute of Psychology, German Sport University Cologne, Cologne, Germany; ^3^Experimental Psychology Unit, Faculty of Humanities and Social Sciences, Helmut Schmidt University/University of the Federal Armed Forces Hamburg, Hamburg, Germany; ^4^Department for Performance and Health (Sports Medicine), Institute for Sport and Sport Science, Technical University Dortmund, Dortmund, Germany

**Keywords:** physical activity, nature, coronavirus disease 2019, stress, psychological health

## Abstract

**Objective:** This cross-sectional questionnaire-based study aims to compare physical activity and nature exposure levels between people living in France and Germany during the lockdown. Furthermore, the secondary aim is to observe the relationship between perceived stress, psychological health, physical activity, and nature exposure in Germany and France during the coronavirus disease 2019 (COVID-19)-related lockdown of April/May 2020.

**Methods:** The study includes 419 participants who have completed the Perceived Stress Scale 10, the World Health Organization Quality of Life**-**BREF, the Godin-Shephard Leisure-Time Physical Activity Questionnaire, the modified Nature Exposure Scale, and complementary questions related to the lockdown period from April 19 to May 11, 2020. Multiple regression models were constructed to evaluate the relationship of nature exposure and physical exercise with overall stress perception and psychological health in France and Germany when considering a broad range of covariates.

**Results:** Exposure to nature during the lockdown (η*p*^2^ = 0.034, *p* < 0.001), amount of physical activity η*p*^2^ = 0.014, *p* < 0.001), and psychological health (η*p*^2^ = 0.041, *p* < 0.001) were greater in German compared with French participants. Godin Index and Nature Exposure Scale total score were both inversely correlated to stress perception and positively correlated to psychological health. The stress and psychological health regression models explained 10% of the results' variance. Physical activity (Godin Index) was a significant for both models. Nature Exposure Scale total score was a significant predictor only for psychological health. When including all significant covariates, the regression models explained 30.7% for the perceived stress and 42.1% for the psychological health total overall variance.

**Conclusion:** Physical activity and nature exposure are significant predictors of psychological health. Even though both variables are associated with stress perception, only physical activity is a significant predictor of stress perception. Our results suggest that physical activity and nature exposure were key factors to go through the lockdown period in France and Germany.

## Introduction

The global spread of coronavirus disease 2019 (COVID-19), potentially leading when contracted to an acute respiratory distress syndrome (ARDS; Li et al., 2020), pushed governments from the whole world to take exceptional decisions to limit the impact of this pandemic and the saturation of world's health systems. In order to limit the spread of infection, some European countries imposed strict isolation quarantine rules upon their citizens. Depending on the government policies, infection levels, and the saturation of health systems, the measures taken by countries varied (World Medical Association, 2020). Between March 17 and May 11, the French government imposed full confinement for its citizens. This required the population to stay at home, non-essential shops were closed, and people had to fill out a very restrictive derogative travel certificate to go outside from their residency (Ministère de l'intérieur français, 2020). In these conditions, the practice of physical activity outside from home had to be individual, not more than 1 h daily, and within a 1-km radius from residency (Ministère de l'intérieur français, 2020). On the contrary, the German government was less restrictive. Indeed, people who lived in Germany during this period were allowed to leave their households to exercise or to merely enjoy some fresh air, but only alone or with one other person.

Physical activity and physical exercise have been used for decades to improve mood and chronic stress regulation in humans of all ages, genders, ethnicities, and cultures (Gladwell et al., 2013; Sjøgaard et al., 2016; Saraulli et al., 2017; Laird et al., 2018). Acute physical activity and exercise have been demonstrated to improve mood and induce adaptive stress resistance through an increase in neuroactive molecules such as serotonin, epinephrine, brain-derived neurotrophic factor, and oxytocin (Strüder and Weicker, 2001; Lafenetre et al., 2011). Chronic physical activity, in particular physical exercise training, leads to metabolic adaptations like decreased cortisol levels (Huang et al., 2013), decreased inflammation (Koliamitra et al., 2019; Małkiewicz et al., 2019), reduced central kynurenine pathway activity (Agudelo et al., 2014; Metcalfe et al., 2018), improved immune function (Phillips and Fahimi, 2018; Rumpf et al., 2021), and decreased permeability of the blood–brain barrier (Małkiewicz et al., 2019). Furthermore, these metabolic changes due to physical activity and exercise offer some benefits on mental health for both clinical and nonclinical populations (Laporte et al., 1985; Deslandes et al., 2009; Javelle et al., 2020; Oberste et al., 2020).

Nature exposure is another parameter suggested to alleviate mood and reduce stress (stress reduction theory and attention restoration theory) (Ulrich, 1981; Kaplan, 1995). Several functional magnetic resonance imaging studies suggest that these restorative effects might be associated with an increased activation of the cuneus, precuneus, and posterior cingulate cortex (Tang et al., 2017; Chang et al., 2021). These brain regions play a key role in dose-dependent stress responses, potentially through extensive connections to the prefrontal and hippocampal regions, which, in turn, project toward the neuroendocrine system (Chang et al., 2021). Pretty and colleagues have observed that nature-related health benefits can be primed when associated with exercise (Pretty et al., 2003, 2005; Barton and Pretty, 2010; Rogerson et al., 2016). To describe potential synergistic health benefits that occur when exercising in nature, the term “green exercise” was endorsed by Pretty et al. in 2003. In a later multi-study analysis, green exercise has been demonstrated to increase moderately mood (*d* = 0.54) and self-esteem (*d* = 0.46) (Barton and Pretty, 2010). Furthermore, Gladwell et al. (2013) have reported in their literature review that green exercise leads to lower levels of perceived exertion, altering physiological functioning including stress reduction, restoring mental fatigue, and improving mood, self-esteem, and perceived health.

Against this backdrop, this questionnaire-based study aims to compare physical activity and nature exposure levels between people living in France and Germany during the lockdown. It was hypothesized that German people would have a greater exercise time and nature exposure than French people. Therefore, German people would have greater psychological health and a lower stress level score than French people. Furthermore, this study aims to quantify the association between physical activity, nature exposure, and stress perception along with psychological health in Germany and France during the lockdown period 2020 (April–May 2020) when controlling for a broad range of covariates.

### Materials and Methods

All procedures were in accordance with the Declaration of Helsinki and were approved by the University Institutional Review Board before data were collected. The corresponding author can provide the full dataset.

### Experimental Design

Participants were recruited through online advertising on the German Sport University website, on social networks (i.e., Facebook and Twitter), and per email in France and Germany using personal and professional networks. No rewards were provided to the participants. Participants completed an online informed consent before engaging in study procedures.

The Perceived Stress Scale (PSS)-10 (Cohen, 1988), the World Health Organization Quality of Life (WHOQoL)-BREF (Harper et al., 1998), the Godin-Shephard Leisure-Time Physical Activity Index (Godin, 2011), the modified Nature Exposure Scale (Wood et al., 2019), and complementary questions related to the lockdown period were administered *via* the online platform Qualtrics from April 19 to May 11‘2020. The survey was available in German and French languages. The inclusion criteria were to be either living in France (and to speak fluently French) or living in Germany (and to speak fluently German) at the time of the survey. To be able to properly assess the situation during the lockdown without having much difference between weeks, participants were asked to consider the past 3 weeks when completing all scales and subsequently did not have to take into account before or the very first lockdown days in their answers. Other control variables (e.g., habits change) recorded for explorative purposes are not presented in this paper because of a lack of space and focus but can be found in Supplementary Material A.

### Sample

#### Sample Size

*A priori* sample size calculations were estimated using the software G^*^Power (version 3.1.9.2). Based on the difference in governmental regulations during the pandemic 2020, at least a small-to-moderate group difference (η*p*^2^ = 0.045) when controlling for two covariates on an ANCOVA was expected for the primary outcomes (physical activity levels and nature exposure between France and Germany). A full sample size of 369 participants was required to meet this purpose. *A priori* calculation for linear multiple regressions, two-tailed, with five predictors (country, Godin Index, Nature Exposure Scale total score, age, and gender) explaining 10% of the general variance (*r*^2^ = 0.100) required 184 participants.

Our recruitment met power requirements with 419 participants included in the analysis.

#### Sample Characteristics

Out of 434 participants, nine who answered one or both catch items incorrectly, and six who brought insufficient care to their answers (*Catch Items and Careless Answers* section) were excluded from the analysis. Missing rate was inferior to 3.5% over all questions in the German and French surveys. Out of the 419 participants included in the final analysis, 13 (10 for Germany and three for France) were non-native speakers but were fluent with the language used and so included in the analysis. Based on the reported zip codes, the majority of participants were located in Rhône-Alpes and North Rhine Westphalia compared with all other regions in France and Germany (Supplementary Material A).

Descriptive characteristics of participants per country are reported in Table 1. Data from 145 French and 274 German participants who completed the battery of questionnaires were available for the analysis. The German sample included 33.6% of males [vs. 48.7% in the global population in 2019 (Statista, 2020a)]. The French sample included 39.3% of males [vs. 48.3% in the global population in 2019 (INSEE, 2020c)]. One German participant reported being diverse and was withdrawn from gender comparison as alone in its category. The French sample included fewer students and more active persons than did the German sample (20–70.3 vs. 40.9–54%; Table 1, *Complementary Questions* section). France reported a greater number of participants living in a village than did Germany (48.3 vs. 17.2%, *Complementary Questions* section, Table 1). The average age was 40.7 years old in the French sample [41.3 in the global population in 2017 (INSEE, 2020a)] and 32.5 years old in the German sample [44.5 in the global population in 2019 (Statista, 2020b)]. The age was significantly different between French and German answers (*p* < 0.010, Table 1).

**Table 1:** Descriptive characteristics of the French and German samples.

	**Groups**	**French version** **(*n* = 145)**	**German version** **(*n* = 274)**
**Categorical variables**			
Gender	Females	88 (60.7%)	181 (66.1%)
	Males	57 (39.3%)	92 (33.6%)
	Diverse	0 (0%)	1(0.4%)
Professional status	Students	29 (20%)	112 (40.9%)
	Retired	11 (7.6%)	6 (2.2%)
	Active	102 (70.3%)	148 (54%)
	Non-active	3 (2.1%)	8 (2.9%)
Chronic disease	Yes	17 (11.7%)	36 (13.1%)
	No	128 (88.3%)	236 (86.9%)
Psychological disorder	Yes	1 (0.7%)	2 (0.7%)
	No	144 (99.3%)	273 (99.3%)
Tutoring	Yes	52 (35.9%)	58 (21.2%)
	No	93 (64.1%)	216 (78.8%)
COVID-19	Suspected	9 (8.3%)	15 (5.4%)
	Diagnosed	3 (2.1%)	1 (0.4%)
Working habits	Change	88 (60.7%)	196 (71.5%)
	No change	18 (12.4%)	46 (16.8%)
	No more work	34 (24.4%)	31 (11.3%)
	n/a	5 (3.4%)	0 (0%)
Living location	Village	70 (48.3%)	47 (17.2%)
	Small city	34 (23.4%)	47(17.2%)
	Big city	41 (28.3%)	180 (65.7%)
Physical activity during the lockdown	Yes	135 (93.3%)	263 (96%)
	No	10 (6.7%)	11 (4%)
**Continuous variables**			
Age (years)		40.7 ± 17.0	32.5 ± 11.9
Minutes per week of physical activity during the lockdown		303.2 ± 211.8	389.2 ± 268.1
Godin Index		38.1 ± 24.4	43.6 ± 24.6

*Note. When the missing rate (n/a) is not listed, it is equal to 0. Results for continuous variables are presented as mean (±standard deviation)*.

*COVID-19, coronavirus disease 2019*.

### Perceived Stress Scale-10 (Cohen, 1988)

The PSS is the most widely used psychological instrument for measuring stress perception (Cohen et al., 1983). It is a measure of the degree to which situations in one's life are appraised as stressful. Items were designed to tap how unpredictable, uncontrollable, and overloaded respondents find their lives (Cohen et al., 1983). The PSS-10 was preferred over the PSS-14 for better reliability, poor loading of four additional items (5, 6, 12, and 13) existing in the PSS-14, and an equivalent validity (Cohen, 1988; Lee, 2012). Participants were asked to respond to all items on a Likert scale (ranging from 1 “never” to 5 “very often”). The validated French and German versions were used (Rolland, 1991; Klein et al., 2016). Overall Cronbach's alpha was.858 (French version, 840; German version, 867).

### World Health Organization Quality of Life-BREF (Harper et al., 1998)

The WHOQoL**-**BREF instrument is a valid and reliable reduced version of WHOQoL-100, which is more convenient for use in large research studies. It comprises 26 items, which measure the following broad domains: physical health, psychological health, social relationships, and environment (Harper et al., 1998). The validated French and German versions were used (Baumann et al., 2010; Burfeind, 2011). Responses to each item are coded from 1 to 5 and averaged per domain. Overall Cronbach's alpha was.847 (French version,798; German version, 823).

### Godin-Shephard Leisure-Time Physical Activity Index (Godin, 2011) and Physical Activity-Related Questions

Participants were asked to judge their average physical activity frequency per week and time per session within the 3 weeks before the questionnaire completion (i.e., during the lockdown period).

The Godin-Shephard Leisure-Time Physical Activity Index is obtained using the following formula: (frequency per week of mild exercise training × 3) + (frequency per week of moderate exercise training × 5) + (frequency per week of strenuous exercise training × 9) (Godin, 2011). The intended cut-point values for the classification scoring are based on the North American public health physical activity guidelines, which are defined as follows: individuals reporting moderate-to-strenuous Leisure Score Index ≥ 24 are classified as active, whereas individuals reporting moderate-to-strenuous Leisure Score Index ≤ 23 are classified as insufficiently active (estimated energy expenditure < 14 kcal/kg/week) (Godin, 2011).

### The Modified Nature Exposure Scale (Wood et al., 2019)

The modified Nature Exposure Scale is a five-item scale designed to assess direct physical and/or sensory contact with the natural environment (Wood et al., 2019). This version was preferred to the four-item scale created by Francis (unpublished) in reason of its better model fit. One item is designed to assess exposure to nature in everyday life, two items to assess exposure outside of everyday environments, and two items assessing nature exposure during physical exercise. Each question on the scale is scored on a 5-point Likert scale (ranging from 1 “high/a great deal” to 5 “low/not much”), with higher scores reflecting greater exposure to nature. A total score was computed as the average of the five items.

French and German versions were translated by the authors and evaluated via confirmatory factor analysis (CFA) before using their results (German and French versions and a short summary from the CFA are available in Supplementary Materials B, C, respectively). Both German and French versions got an acceptable fit (one-factor structure) but should be retested independently from the lockdown period. Overall Cronbach's alpha was.811 (French version, 847; German version, 811).

### Complementary Questions

Several questions were added to the previously mentioned questionnaires to relate to the current pandemic and assess potential covariates of stress and psychological health that could influence our results.

Participants were asked their age, gender [i.e., male, female, and diverse), and status (i.e., student, retired, active, and non-active—based on the definitions of the French National Institute of Statistics and Economics (INSEE, 2020b)]. They were asked to report if they had any other persons under their responsibility (e.g., children and elders) at home; if their work habits changed; if they were living in a village, a small city, or a large city; and the zip code of their residency during the lockdown period.

Participants were asked to report if they were suffering from any chronic diseases and, if so, what specific type. These open-ended responses were then categorized as psychological or physical disorders by the experimenters. Participants had to report if they had been suspected and/or diagnosed to be suffering from COVID-19. These participants were kept in the analysis, but this variable was considered as a potential covariate. This variable was considered binary (suspected or diagnosed vs. healthy) and on three levels (suspected vs. diagnosed vs. healthy).

Finally, the day of questionnaire completion was recorded to control for the duration of the lockdown period within the 3 weeks where our questionnaire was offered online.

### Catch Items and Careless Answers

Two catch items were strategically placed along the questionnaire. These catch items (please select “I agree”) were presented as being catch items (“Checking for random answers”) to avoid that attentive responders consider these items as mistakes or parts of the psychological assessment. They were later used to identify and exclude random answers. Methods explained by Meade and colleagues to reduce, identify, and remove careless response were also applied to this questionnaire (Meade and Craig, 2012). First names, acronyms, or pseudonyms (participants' choice) were used to interact with participants. At the end of the questionnaire, participants had to evaluate the level of care they brought to their answers on a scale going from 1 (almost no care) to 5 (full care). Participants reporting a level of care below 3 (moderated care) were excluded from the analysis. Participants were also asked if they were considering the quality of their answers sufficient to enter in our analysis. Participants answering no were excluded from the analysis.

### Statistics

#### Generals

Statistical analyses were conducted using SPSS software, version 23.0 (IBM, Armonk, New York) and R, version 1.2.1335. Data were first winsorized at 1% and 99% (±2.58 standard deviations) for both countries independently. Based on the central limit theorem applied to large numbers, our samples' sampling distribution was normal. Nevertheless, all variables were checked for linearity (*via* quantile–quantile plot, and histogram of standardized residuals), skewness, and kurtosis. If these assumptions were not met, data were corrected with the appropriated transformation. Thus, times of exercise before and during the lockdown period were square root corrected and then met the linearity assumptions. Descriptive characteristics were compared between countries using *t*-tests. Questionnaire results considering potential covariates were compared between countries using analysis of covariance, ANCOVA. “Professional status,” “age,” and “living location” were the most differently distributed variables between our samples. “Tutoring” and “working habits” distributions also moderately differed between our samples. This is very likely due to a large overlap with the previously mentioned variables. Subsequently, “professional status,” “age,” and “living location” were considered as covariates in the univariate analyses. Relationships between variables of the full dataset were evaluated with bivariate correlations and regression models (*Multiple Linear Regression Models* section). Bonferonni alpha correction was applied family-wise for comparison and correlation analyses. So physical exercise indices (time of exercise and Godin Index) were considered significant at *p* < 0.025.

#### Multiple Linear Regression Models

A correlational table and a categorical analysis were constructed to introduce potential links between dependent variables and covariates. Only the significant covariates detected were then used to construct the multiple regression models (Supplementary Material A).

The outcome values from multiple regression models were always continuous (PSS-10 and WHOQoL-BREF psychological health levels), and the main predictor for each model was either binary or continuous (Field, 2013). All predictors were tested for multicollinearity (variance inflation factor and tolerance values—values accepted were respectively <2, and >0.2), independence (Durbin Watson test—results accepted were between 1 and 3), and linearity (graphically *via* quantile–quantile plot, scatterplot, and histogram of studentized residuals) (Bowerman and O'Connell, 1990). Predictors and models met all the previously mentioned assumptions.

*Model 1*. Differences in perceived stress levels between France and Germany were the first interest, so the variable “country” was chosen as the main predictor. Other independent predictors were selected based on literature and then on results from univariate analysis. Godin Index scores are supposed to be better fitness indices than simple exercise durations and thus were selected as exercise predictor. Nature Exposure Scale total score, age, gender, and professional status were also included to the model to account for differences in the sampling distribution from each survey.

*Model 2*. A more complete model applied to all participants was created to better understand participants' stress levels independently from the country of provenance. Additional variables potentially impacting stress levels were added to the model, while the variable “country” was withdrawn. All predictors were still independent (e.g., chronic disease could not have been included as a predictor because it was already represented by the continuum scores existing within the physical health domain). All domains from the WHOQoL-BREF (apart from psychological health), professional status, age, gender, Godin Index, and Nature Exposure Scale total score were considered as potential covariates.

*Model 3*. Differences in psychological health levels between France and Germany were the second interest, so the variable “country” was chosen as main predictor for the initial model. Godin Index, Nature Exposure total score, professional status, and gender were the other predictors.

*Model 4*. A more complete model was created to better understand participants' psychological health levels independently from the country of provenance. Additional variables potentially impacting psychological health were added to the model, while the variable “country” was withdrawn. All predictors were still independent. All the other domains from the WHOQoL-BREF, professional status, gender, Godin Index, and Nature Exposure Scale total score were considered as potential covariates.

## Results

When accounted for the effects of age, professional status, and living location, participants from Germany reported doing more physical exercise (η*p*^2^ = 0.014, *p* < 0.05) during the lockdown than participants living in France. Godin Index scores were not different between countries (η*p*^2^ = 0.002, *p* = 0.335). The average Godin Index scores for both countries were largely above the cutoff score of 24 to be considered as being active (40.0 vs. 42.7).

### Questionnaire Result

The differences between groups from each questionnaire are presented in Table 2. When controlled for age, professional status, and living location, participants from Germany had significantly greater stress perception levels (η*p*^2^ = 0.014, *p* < 0.050). WHOQoL-BREF psychological health domain was significantly greater for the participants from Germany (η*p*^2^ = 0.041, *p* < 0.001). The Nature Exposure total score was greater for participants from Germany when controlled for the place participants were living in (η*p*^2^ = 0.034, *p* < 0.001).

**Table 2:** Comparisons between France and Germany in questionnaire results accounting for the effect of age, professional status, and living location.

**Questionnaire**		**Significant covariates**	**Effect size (*ηp^**2**^*), *p* value**	**Adjusted means** ± **Std. error**	
				**France**	**Germany**
PSS-10		Age	0.014, *p* < 0.050	2.51 ± 0.06	2.68 ± 0.04
Nature exposure total score		Age	0.034, *p* < 0.001	3.32 ± 0.08	3.71 ± 0.06
		Living location			
WHOQoL-BREF	Physical health	Age	0.009, *p* = 0.155	12.18 ± 0.16	12.59 ± 0.11
	Psychological health	–	0.041, *p* < 0.001	13.34 ± 0.22	14.42 ± 0.15
	Social relationships	–	0.000, *p* = 0.953	14.34 ± 0.29	14.37 ± 0.20
	Environment	–	0.007, *p* = 0.083	15.86 ± 0.17	16.01 ± 0.12

*Note. PSS-10, Perceived Stress Scale 10; WHOQoL, World Health Organization Quality of Life*.

Exercise time during the pandemic and Godin Index scores were inversely correlated to PSS-10 levels (*r*'s > – 0.136, *p*'s < 0.01), showing a negative association between physical activity and perceived stress levels (Table 3). Moreover, the nature exposure score also negatively correlated to stress levels (*r* = −0.128, *p* < 0.010). Positive correlations were observed between psychological health and physical activity indices as well as nature exposure score (*r*'s > 0.180, *p*'s < 0.001).

**Table 3:** Correlations table between physical activity indices, Nature Exposure Scale total scores, and questionnaire results.

	**DOM1**	**DOM2**	**DOM3**	**DOM4**	**Exercise time**	**Godin Index**	**Nature**	**Age**	**Completion date**
PSS-10— Perceived stress	−0.471^***^ 0.000	−0.578^***^0.000	−0.341^***^ 0.000	−0.350^***^0.000	−0.140^**^ 0.005	−0.136^**^0.007	−0.128^**^ 0.009	−0.204^***^0.000	0.000 0.997
DOM1— Physical health		0.551^***^ 0.000	0.336^***^ 0.000	0.469^***^0.000	0.140^**^ 0.005	0.164^**^0.001	0.165^*^ 0.001	0.158^***^0.001	0.000 0.992
DOM2— Psychological health			0.427^***^ 0.000	0.439^***^0.000	0.180^***^ 0.000	0.192^***^0.000	0.195^***^ 0.000	0.0240.623	−0.073 0.135
DOM3— Social relationship				0.302^**^0.000	0.053 0.293	0.0500.323	0.075 0.128	0.0700.151	0.010 0.838
DOM4— Environment					0.120^*^ 0.017	0.193^***^0.000	0.202^***^0.000	0.0420.395	−0.043 0.382
Exercise time during the pandemic						0.749^***^0.000	0.268^***^ 0.000	−0.0120.809	−0.113^*^ 0.024
Godin Index							0.199^***^ 0.000	−0.209^***^0.000	−0.139^**^ 0.005
Nature Exposure Score								0.199^***^0.000	−0.047 0.344
Age									0.220^***^ 0.000

*Note. PSS-10, Perceived Stress Scale 10*.

* *p < 0.050*;

** *p < 0.010*;

*** *p < 0.001*.

The completion date was inversely correlated to the time of exercise during the lockdown and the Godin Index (*r*'s > – 0.113, *p*'s < 0.050) and positively correlated to age (*r* = 0.220, *p* < 0.001) but did not show any association with perceived stress and psychological health.

Univariate analysis of the impact of each categorical covariate of PSS-10 and psychological health levels was as well-realized (Supplementary Material A). Only gender and professional status reached significance (Supplementary Material A).

### Multiple Linear Regression Models

Multiple linear regression models results are reported in Table 4.

**Table 4:** Multiple linear regression model results table.

**Model**	***R*^2^—*p* value**	**Variable**	**Estimate (β)**	**Std. error**	***p* value**
1	Multi. *R*^2^ = 11.3% Adj. *R*^2^ = 9.9%	Intercept	3.00	0.207	*p* < 0.001
	*p* < 0.001	Country	0.187	0.071	*p* < 0.010
		Gender	0.029	0.066	*p* = 0.659
		Godin Index	−0.005	0.001	*p* < 0.001
		Nature Exposure	−0.041	0.035	*p* = 0.245
		Status	−0.033	0.024	*p* = 0.163
		Age	−0.008	0.002	*p* < 0.010
2	Multi. *R*^2^ = 32.1% Adj. *R*^2^ = 30.7%	Intercept	5.687	0.79	*p* < 0.001
	*p* < 0.001	DOM1	−0.106	0.019	*p* < 0.001
		DOM3	−0.038	0.009	*p* < 0.001
		DOM4	−0.056	0.017	*p* < 0.010
		Nature Exposure	0.010	0.031	*p* = 0.740
		Godin Index	−0.003	0.001	*p* < 0.050
		Gender	0.055	0.056	*p* = 0.330
		Status	–.024	0.021	*p* = 0.264
		Age	−0.007	0.002	*p* < 0.010
3	Multi. *R*^2^ = 11.2% Adj. *R*^2^ = 10.1%	Intercept	10.998	0.562	*p* < 0.001
	*p* < 0.001	Country	1.004	0.262	*p* < 0.001
		Gender	−0.482	0.246	*p* = 0.051
		Godin Index	0.018	0.005	*p* < 0.001
		Nature Exposure	0.409	0.129	*p* < 0.010
		Status	0.171	0.083	*p* < 0.050
4	Multi. *R*^2^ = 43.2% Adj. *R*^2^ = 42.1%	Intercept	0.622	0.956	*p* = 0.669
	*p* < 0.001	DOM1	0.504	0.064	*p* < 0.001
		DOM3	0.196	0.032	*p* < 0.001
		DOM4	0.232	0.060	*p* < 0.001
		Nature Exposure	0.197	0.104	*p* < 0.050
		Godin Index	0.009	0.004	*p* < 0.050
		Gender	−0.164	0.193	*p* = 0.395
		Status	0.023	0.066	*p* = 0.730

*Multi., Multiple; Adj., Adjusted*.

#### PSS-10—Perceived Stress Levels

*Model 1*. Out of the six predictors, Nature Exposure Scale total score (*β* = – 0.041, *p* = 0.245), professional status (*β* = – 0.033, *p* = 0.163), and gender (women compared with men, *β* = −0.029, *p* = 0.659) were not significant. The model explained 9.9% (*p* < 0.001) of the overall variance where country (Germany compared with France, *β* = – 0.187, *p* < 0.010), Godin Index (*β* = – 0.005, *p* < 0.001), and age (*β* = – 0.008, *p* < 0.010) were all negatively associated with stress levels.

**Figure 1:**
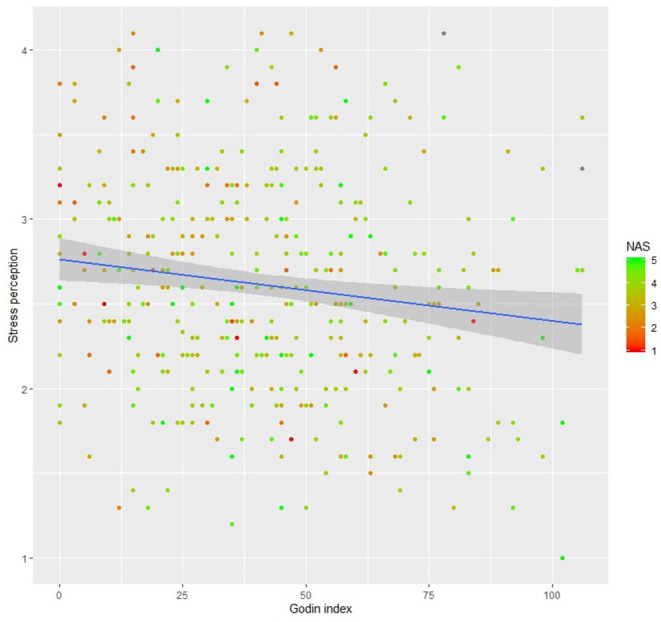
Perceived Stress Scale 10 (PSS-10) multiple regression illustration with only two predictors: Godin Index and Nature Exposure total Score (NAS). Missing values in the NAS are visualized in gray. The regression line (in blue) is presented with 95% of confidence interval (gray area).

*Model 2*. The regression model applied to the full sample explained 30.7% (*p* < 0.001) of the overall variance. Nevertheless, professional status (*β* = – 0.024, *p* = 0.264), gender (women compared with men, *β* = 0.055, *p* = 0.330), and Nature Exposure Scale total score (*β* = 0.010, *p* = 0.740) were not significant predictors. Physical health (DOM1, *β* = – 0.106, *p* < 0.001), social relationships (DOM3, *β* = – 0.038, *p* < 0.001), environment (DOM4, *β* = –0.056, *p* < 0.010), Godin Index (*β* = – 0.003, *p* < 0.050), and age (*β* = – 0.007, *p* < 0.010) were associated with stress levels. The relation between stress perception, nature exposure, and Godin Index is illustrated in Figure 1.

#### World Health Organization Quality of Life—BREF—Psychological Health

*Model 3*. Out of the five predictors, only gender (women compared with men, *β* = −0.482, *p* = 0.051) was not significant. This model explained 10.1% (*p* < 0.001) of the overall variance where country (Germany compared with France, *β* = −1.005, *p* < 0.001), Godin Index (*β* = 0.018, *p* < 0.001), nature exposure (*β* = 0.409, *p* < 0.010), and professional status (*β* = 0.170, *p* < 0.050) were all positively associated with psychological health.

**Figure 2:**
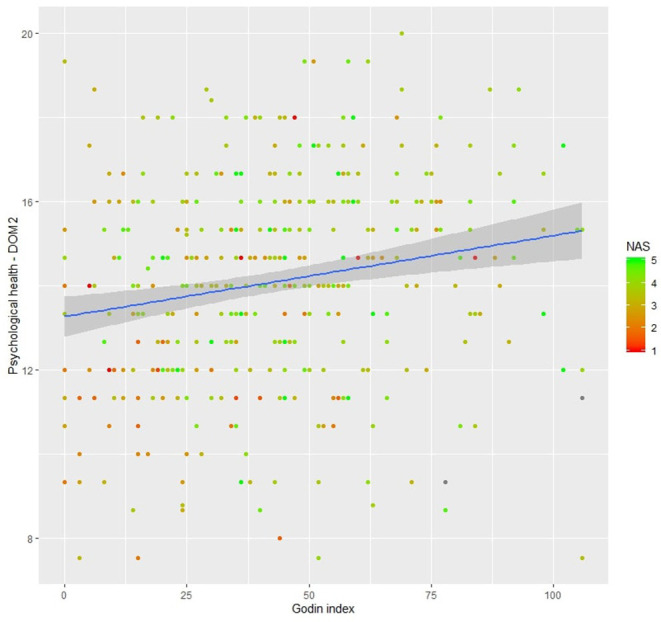
Psychological health multiple regression illustration with only two predictors: Godin Index and Nature Exposure total Score (NAS). Missing values in the NAS are visualized in gray. The regression line (in blue) is presented with 95% of confidence interval (gray area).

*Model 4*. The resulting model explained 42.1% (*p* < 0.001) of the overall variance. Nevertheless, professional status (*β* = 0.023, *p* = 0.730) and gender (women compared with men, *β* = – 0.164, *p* = 0.395) were not significant predictors. Physical health (*β* = 0.504, *p* < 0.001), social relationship (*β* = 0.196, *p* < 0.001), environment (*β* = 0.232, *p* < 0.001), Godin Index (*β* = 0.009, *p* < 0.050), and Nature Exposure Scale total score (*β* = 0.197, *p* < 0.050) were positively associated with psychological health. The relation between psychological health, nature exposure, and Godin Index is illustrated in Figure 2.

## Discussion

This questionnaire-based study aimed to compare physical activity and nature exposure levels along with stress perception and psychological health in German and French samples during the lockdown period (April–May 2020). Furthermore, the association of physical activity and nature exposure with stress perception and psychological health was evaluated through multiple linear regression models.

Participants from Germany reported doing significantly more physical activity during the lockdown period than participants from France (Table 1) and consequently had a higher Godin Index score that was, nonetheless, not significant after controlling for the effect of age, professional status, and living location. The effect of the French complete lockdown on outdoor activities appeared even more clearly when considering Nature Exposure Scale total scores. Even after controlling for living location, Nature Exposure Scale total scores were higher in German participants (small-to-moderate effect size; Table 2). These results confirmed our hypothesis. During the lockdown, the German sample was more outside and did more physical activity than the French sample. Considering the reduced amount of time allowed outside (i.e., 1 h per day), the additional effort to go outdoors do some activities (i.e., to always have a derogative travel certificate) vs. no limitation of time but of escort in Germany, these results appeared logical. Furthermore, differences of psychological health levels in favor of the German participants (small-to-moderate effect size) were detected. Nonetheless, German participants also reported higher perceived stress than French participants did (small effect size).

Going further in our analysis, we have tested the detected differences in regression models. Together with the variable “country,” physical activity and nature exposure were significant predictors of psychological health levels and responsible for a small-to-moderate part of the total variance (10%). The positive relationship displayed in the regression model (and in the correlations table) between psychological health, physical activity, and nature exposure is in line with the scientific literature already showing convincing evidence from numerous studies (Plante and Rodin, 1990; Atlantis et al., 2004). The interaction between physical exercise and mental health results from multiple factors (Plante and Rodin, 1990). For example, increased neurotransmission of norepinephrine, serotonin, and dopamine following exercise has been shown to improve mood, muscle potential, and tension release and to give a sense of mastery control and self-sufficiency (Plante and Rodin, 1990). Furthermore, chronic exercise has been shown to improve immune functions as well as kynurenine pathway regulation that are indirect regulators of mood and psychological health (Won and Kim, 2016; Metcalfe et al., 2018; Małkiewicz et al., 2019). When considering exposure to nature (physical and/or sensory contact with the natural environment), previous studies have shown that the latter can improve mental well-being, attention, and mood. Indeed, nature health-related benefits have been acknowledged for a very long time (Hartig and Marcus, 2006). When including significant covariates, and applied to the full sample (without countries comparison), the regression model created for psychological health explained more than 40% of the overall variance. Nature Exposure Scale total score and Godin Index were determined as being significant predictors of psychological health, alongside with physical health (DOM1), environment (DOM3), and social relationships (DOM4). For decades, the scientific community has recognized the association between physical and psychological health (Plante and Rodin, 1990). Nevertheless, our results are also among the first to show that nature deficiency mostly exists in inactive people and psychological health (Figure 2; Godin Index < 25—DOM2 ≤ 14).

When considering stress perception, the regression model also explained a small-to-moderate part of the total variance (10%). Nevertheless, only country provenance, Godin Index, and age were significant predictors. After including covariates and when applied to the full sample (without countries comparison), the regression model explained 30% of the overall variance. In line with the literature, physical health (DOM1), environment (DOM3), social relationships (DOM4), Godin Index, and age were determined as significant predictors. These results not only confirm already acknowledged ties (Plante and Rodin, 1990; Talley et al., 2012; Campos et al., 2014) but also show that physical activity is a direct predictor of perceived stress level (Figure 1). Indeed, a number of randomized controlled trials have shown that exercise training is an effective method for improving stress symptoms and quality of life (Weyerer and Kupfer, 1994; Atlantis et al., 2004; Imayama et al., 2011). Exercise blocks the effect of psychological stress on cardiac reactivity (Hamer et al., 2006) and dampens stressor-evoked increases in stress hormones (Stults-Kolehmainen and Sinha, 2014). Even though negatively correlated to stress perception, Nature Exposure Scale total score did not appear as a significant predictor of PSS-10 scores. Our results suggest that nature exposure is a more consistent predictor for psychological health than for stress perception.

Despite its novelty and strengths, several limitations exist. Firstly, the initial difference suggesting that German participants may have been more stressed than French (small effect size) was unexpected. This is especially surprising considering the higher levels of physical activity and nature exposure in the German survey. Regardless, univariate analyses were controlled for variables that were differently distributed between our samples (i.e., age, living location, and professional status). It is possible that other parameters may have affected our results. An explanation for this difference could be given by uncontrolled covariates, like dominant personality traits [e.g., neuroticism is associated with stress reactivity (Lahey, 2009)], that were not evaluated given the trade-off between questionnaire length and sample size aims. Secondly, the recruitment in Germany was partly conducted at the German Sport University campus hosting many sport students, whereas no professional sport sciences network was used in France. This may have affected the overall physical activity indices of the German sample and could be another explanation of the difference in physical exercise time during the lockdown period between the German and French samples. Thirdly, small effect size results in the univariate analyses' (i.e., stress perception and physical activity time during the lockdown) lack of power (46%, post hoc sensitivity analysis for ANCOVA with three covariates and η*p*^2^ = 0.014). The power may have been even more reduced because of the unbalance of the German and French samples leading to unequal variances. Nevertheless, this was not detected by Levene's tests. Fourthly, even though the best methods to extract representative values data were used, home-based questionnaires are, still, less accurate than interviews or more direct physical exercise measurements (e.g., tracking device and actigraphy). Finally, in this context, it is important to consider in the interpretation of the present study that not all physical activities (e.g., house work) correlate with good health (Lawlor et al., 2002). Nevertheless, these activities may be performed in a health-enhancing manner, like physical exercise, if relevant muscle groups are involved, with sufficient intensity and appropriate recovery.

## Conclusions

In conclusion, the results of this study captured the impact of the lockdown decisions on physical activity, nature exposure, and consequently on psychological health and perceived stress levels. Regression models showed that nature exposure and physical activity are both predictors of psychological health. Physical activity was a predictor of perceived stress levels as well. Our results suggest that physical activity and nature exposure have represented a key factor to go through the lockdown period in France and Germany. Considering previous literature and these new findings, nature exposure and physical activity should be considered more systematically as moderators of psychological health and potentially as complements or alternatives to pharmacological treatments for mood-related disorders and syndromes.

## Data Availability Statement

The raw data supporting the conclusions of this article will be made available by the authors, without undue reservation.

## Ethics Statement

This study involving human participants was reviewed and approved by German Sport University Institutional Review Board. The participants provided an online informed consent to participate in this study.

## Author Contributions

FJ, TH, SL, and PZ did the conceptualization of the study. FJ did the project administration. FJ did the data curation. FJ did the data analysis. FJ, TH, AM, SL, and PZ wrote, reviewed, and edited the manuscript. AM proofread the manuscript. All authors contributed to the article and approved the submitted version.

## Conflict of Interest

The authors declare that the research was conducted in the absence of any commercial or financial relationships that could be construed as a potential conflict of interest. The handling editor declared a past co-authorship with one of the author SL.
