# Supplementary material for: The Importance of Nature Exposure and Physical Activity for Psychological Health and Stress Perception: Evidence From the First Lockdown Period During the Coronavirus Pandemic 2020 in France and Germany
Source: Front Psychol. 2021 Mar 4;12:623946. doi: 10.3389/fpsyg.2021.623946 (PMC7969516; doi:10.3389/fpsyg.2021.623946)
Supplement: Supplementary file 1 [file Table_1.docx]

Table 1

*Habits changes for people living in France versus the ones living in Germany.*

| **Changes** | **France** (n=145) | | **Germany** (n=274) | |
| --- | --- | --- | --- | --- |
| *Diet quality* |  | 5.7 (2.0) |  | 5.6 (2.2) |
| *Sleep quality* |  | 4.8 (2.2) | n/a: 1 | 5.1 (2.2) |
| *Caffeine consumption* | YES: 91  NO: 54 | 4.9 (1.8) | YES: 216  NO: 58 | 5.5 (1.8) |
| *Nicotine consumption* | YES: 48  NO: 97 | 5.0 (2.8) | YES: 31  NO: 243 | 4.8 (2.5) |
| *Alcohol consumption* | YES: 118  NO: 26  n/a: 1 | 4.7 (2.4) | YES: 204  NO: 70 | 4.7 (2.6) |
| *Drugs use* | YES: 25  NO: 119  n/a: 1 | 3.3 (3.3) | YES: 27  NO: 247 | 5.0 (2.0) |

*Notes: Data are presented as mean (standard deviation). n/a= not available.*

As displayed in Table 1, habits changes within the three weeks preceding the questionnaire completion were relatively comparable between French and German answers. Nevertheless, significant differences in drugs use change (*d=.62, p<.05*) and in caffeine consumption change (*d=.33, p<.05*) were detected. No or very minor changes appeared for sleep quality, nicotine use and alcohol consumption (see Table 2). In average, both people living in France and Germany reported a small increase in diet quality (*95%CI 5.4 to 5.8* - see Table 2).

Table 2

*Univariate analyses from potential categorical covariate effects on PSS-10 and WHOQoL psychological health – DOM2 levels.*

| **Categorical covariates** | **PSS10** | **WHOQoL DOM2** |
| --- | --- | --- |
| **Gender**  Male (n=149)  Females (n=269) | ***d=*.48 - *p<.*05**  *2.42 (.63)*  *2.72 (.64)* | ***d=.*34 *- p<.*001**  *14.58 (2.41)*  *13.76 (2.45)* |
| **Status**  Students (n=141)  Retired (n=17)  Actives (n=250)  Non-actives (n=11) | ***ƞp²*=.050 – *p<.*001**  *2.79 (.66)*  *2.33 (.62)*  *2.52 (.62)*  *2.92 (.63)* | p=.26  *13.81(2.81)*  *13.67 (2.07)*  *14.23 (2.24)*  *13.33 (3.16)* |
| **Tutoring**  Yes (n=110)  No (n=309) | p=.47  *2.65 (.63)*  *2.60 (.66)* | p=.20  *13.78 (1.97)*  *14.13 (2.62)* |
| **Exercise type**  Physical Activity [PA] (n=44)  Physical Exercise and PA (n=354)  No training (n=12) | p=.35  *2.60 (.65)*  *2.64 (.74)*  *2.88 (.58)* | p=.124  *13.38 (2.63)*  *14.14 (2.41)*  *13.67 (2.67)* |
| **Working habits**  Yes (n=284)  No (n=64)  I cannot work anymore (n=65) | p=.17  *2.65 (.64)*  *2.51 (.69)*  *2.55 (.71)* | p=.55  *14.09 (2.44)*  *14.19 (2.51)*  *13.73 (2.66)* |
| **COVID suspicion/diagnostic**  Yes (n=28)  No (n=391) | p=.94  *2.61 (.55)*  *2.62 (.66)* | p=.64  *14.25 (2.28)*  *14.07 (2.48)* |
| **Location**  Village (n=117)  Small city (n=81)  Big city (n=221) | p=.66  *2.60 (.70)*  *2.57 (.71)*  *2.62 (.66)* | p=.54  *13.83 (2.14)*  *14.14 (2.47)*  *14.12 (2.63)* |
| **Health-related**  Yes (n=118)  No (n=289) | p=.69  *2.53 (.63)*  *2.66 (.65)* | p=.27  *14.26 (2.41)*  *13.96 (2.51)* |
| **Chronic disease**  Yes(n=53)  No (n=366) | p=.64  2.58 (.57)  2.62 (.67) | p=.90  14.0 (2.43)  14.05 (2.47) |

*Notes. Results are presented as mean (standard deviation). ƞp²= partial eta squared. d= Cohen’s d effect size.*


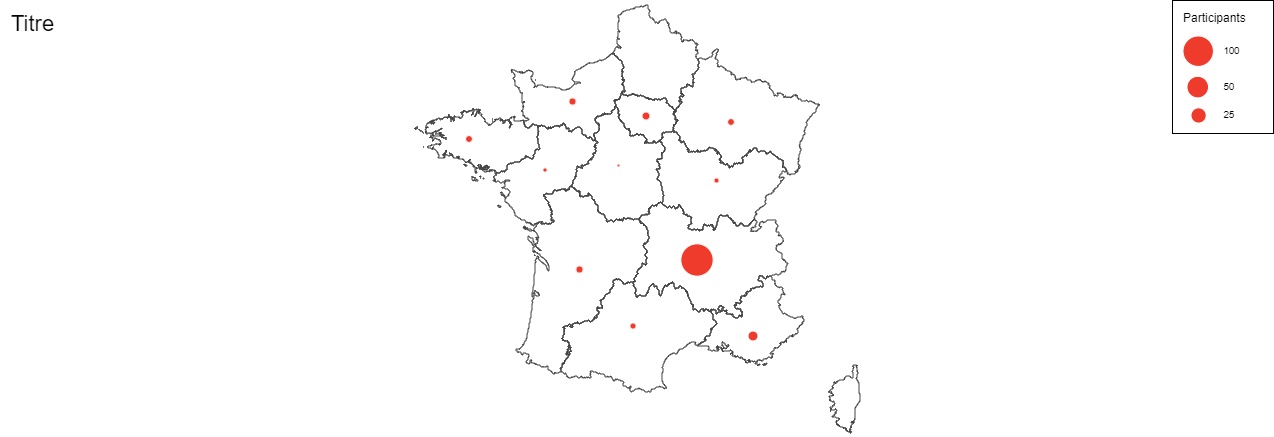

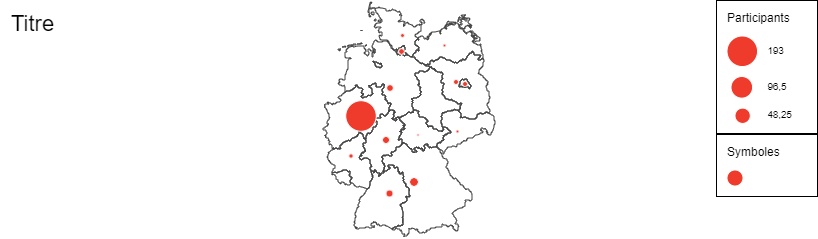

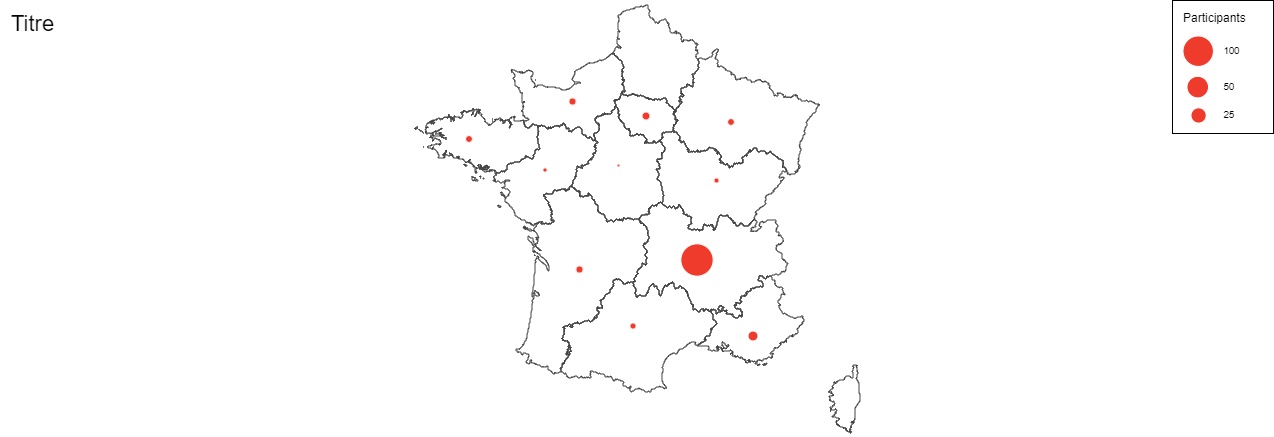


Figure 1: Participants reported zip codes during the lockdown period
