# Supplementary material for: The Importance of Nature Exposure and Physical Activity for Psychological Health and Stress Perception: Evidence From the First Lockdown Period During the Coronavirus Pandemic 2020 in France and Germany
Source: Front Psychol. 2021 Mar 4;12:623946. doi: 10.3389/fpsyg.2021.623946 (PMC7969516; doi:10.3389/fpsyg.2021.623946)
Supplement: Supplementary file 2 [file Table_2.docx]

The original English modified Nature Exposure Scale was taken from (Wood et al., 2019).

Wood, C., Barron, D., Smyth, N., 2019. The current and retrospective intentional nature exposure scales: Development and factorial validity. Int. J. Environ. Res. Public Health 16. https://doi.org/10.3390/ijerph16224443

## Nature Exposure Scale French version

Nous sommes intéressés à votre exposition à la nature, tant dans votre vie quotidienne que dans vos activités, et lorsque vous faites des excursions en dehors de votre environnement quotidien. Nous sommes également intéressés par votre exposition aux environnements naturels pendant votre activité physique. Veuillez répondre aux questions suivantes afin de refléter votre niveau actuel d'exposition aux environnements naturels et votre participation à des activités physiques dans ces environnements.
 Ces "environnements naturels" peuvent se trouver dans des zones urbaines (ex: parcs) ou rurales. Ils peuvent inclure des éléments tels que des plantes, des animaux, une topographie variée (ex: collines, montagnes, déserts, plages, marais), des cours / plans d'eau naturels (ex: rivières, ruisseaux, lacs, étangs et océan). L’environnement naturel contraste avec l'environnement dit "bâti" des maisons, des bâtiments, des routes et de toutes les autres structures de ce type créées par l'homme.  **Dans le cadre de ces questions, votre quotidien se restreint aux trois dernières semaines.**

**L'exposition à la nature dans votre vie quotidienne et dans votre environnement au cours des trois dernières semaines**

1) Dans quelle mesure remarquez-vous les environnements naturels dans votre vie quotidienne ?

|  | Peu  1 | 2 | Assez  3 | 4 | Beaucoup  5 |
| --- | --- | --- | --- | --- | --- |
|  |  |  |  |  |  |

**Exposition à la nature lors d'excursions en dehors des environnements de la vie quotidienne au cours des trois dernières semaines** Ces questions portent sur votre niveau d'exposition à la nature lorsque vous êtes en dehors de votre vie quotidienne. Cela comprend les voyages que vous faites pendant votre temps libre (ou occasionnellement dans le cadre de vos études, de votre travail ou de vos activités sociales) dans des environnements riches en nature dans des zones urbaines, rurales ou sauvages. Ces environnements peuvent être des lieux où vous vous rendez une fois par semaine, ou moins fréquemment, soit avec l’objectif de vous retrouver dans cet environnement naturel, ou bien pour un autre objectif.

2) Veuillez évaluer la fréquence (combien de fois) d'exposition à des environnements riches en nature à l'extérieur votre environnement quotidien :

- Tous les jours
- 3 fois par semaine
- 1 fois par semaine
- 1 fois toutes les deux semaines
- Pas depuis trois semaines

3) Dans quelle mesure avez-vous tenu compte de la nature dans ces environnements ?

|  | Peu  1 | 2 | Assez  3 | 4 | Beaucoup  5 |
| --- | --- | --- | --- | --- | --- |
|  |  |  |  |  |  |

**Exposition à la nature pendant l'activité physique au cours des trois dernières semaines**Ces questions portent sur votre niveau d'exposition à la nature lorsque vous vous engagez dans des activités physiques. L'activité physique en milieu naturel peut comprendre des activités comme par exemple la marche, le fitness dans un parc ou jardin, le jardinage, l’escalade, le jogging ou encore le vélo (liste non-exhaustive). Ces activités physiques peuvent être pratiquées dans le cadre ou coïncidant avec une activité quotidienne, ou être une période d'exercice planifiée. Ils peuvent avoir lieu dans les zones urbaines, rurales ou sauvages.

4) Veuillez évaluer la fréquence (combien de fois) à laquelle vous pratiquez une activité physique dans des environnements riches en nature pendant ces trois dernières semaines:

- Tous les jours
- 3 fois par semaine
- 1 fois par semaine
- 1 fois toutes les deux semaines
- Pas depuis 3 semaines

5) Dans quelle mesure avez-vous tenu compte de la nature lorsque vous pratiquiez une activité physique ?

|  | Peu  1 | 2 | Assez  3 | 4 | Beaucoup  5 |
| --- | --- | --- | --- | --- | --- |
|  |  |  |  |  |  |

## Nature Exposure Scale German version

Wir interessieren uns für das Ausmaß Ihrer Exposition gegenüber Natur und natürlichen Umgebungen. Hierbei interessiert uns sowohl wie viel Zeit Sie während Ihres Alltagslebens und Ihrer Alltagsaktivitäten in der Natur verbringen, als auch das Ausmaß der Zeit, die Sie im Rahmen von Ausflügen in der Natur verbringen. Wir interessieren uns weiterhin dafür, inwiefern Sie natürliche Umgebungen für körperliche Aktivitäten nutzen. Bitte beantworten Sie die folgenden Fragen, um das Ausmaß anzugeben, in dem Sie Zeit in natürlichen Umgebungen verbringen bzw. in der natürlichen Umgebungen sportlich aktiv sind.   Diese "natürlichen Umgebungen" können sich in städtischen (z.B. Parks) oder ländlichen Gebieten befinden. Sie können Elemente wie Pflanzen, Tiere, Naturtopographie (z.B. Hügel, Berge, Wüsten, Strände, Sümpfe), natürliche Wasserwege und Wasserlandschaften (z.B. Flüsse, Bäche, Seen, Teiche und Ozeane) umfassen. Dies steht im Gegensatz zu der sogenannten "gebauten Umgebung" von Häusern, Gebäuden, Straßen und allen anderen derartigen, von Menschenhand geschaffenen, Strukturen.   **Die folgenden Fragen beziehen sich auf Ihren Tagesablauf in den letzten drei Wochen.**

**Umgang mit der Natur in Ihrem täglichen Leben und Ihrer Umwelt**

1) Inwieweit nehmen Sie natürliche Umgebungen in Ihrem täglichen Leben wahr:

|  | Nicht sehr viel  1 | 2 | Ziemlich viel  3 | | 4 | Viel  5 |
| --- | --- | --- | --- | --- | --- | --- |
|  |  |  |  |  | |  |

**Exposition gegenüber der Natur auf Exkursionen außerhalb der alltäglichen Lebensumgebungen** Diese Fragen konzentrieren sich auf den Grad Ihrer Exposition gegenüber der Natur, wenn Sie von Ihrem täglichen Umweltleben abwesend sind. Dazu gehören Reisen, die Sie in Ihrer Freizeit (oder gelegentlich im Rahmen Ihres Studiums, Ihrer Arbeit oder sozialer Aktivitäten) in natürliche Umgebungen in städtischen, ländlichen oder wilden Gebieten unternehmen. Dabei kann es sich um Orte handeln, die Sie einmal pro Woche oder seltener besuchen, entweder mit dem ausdrücklichen Ziel, sich in der natürlichen Umgebung aufzuhalten, oder für einen anderen Hauptzweck.

2) Beurteilen Sie bitte in Ihrer Umgebung und bei Ihren täglichen Aktivitäten zu Hause, auf Reisen und bei der Arbeit den Grad Ihrer Exposition gegenüber natürlichen Umgebungen:

- Täglich
- 3 Mal pro Woche
- Einmal pro Woche
- einmal alle zwei Wochen
- Nicht seit drei Wochen

3) Wie viel Aufmerksamkeit widmen Sie der Natur in diesen Umgebungen?

|  | Nicht sehr viel  1 | 2 | Ziemlich viel  3 | 4 | Viel  5 |
| --- | --- | --- | --- | --- | --- |
|  |  |  |  |  |  |

**Exposition gegenüber der Natur bei körperlicher Aktivität** Bei diesen Fragen geht es um den Grad Ihrer Exposition gegenüber der Natur, wenn Sie sich körperlich betätigen. Körperliche Aktivität in der natürlichen Umgebung kann Aktivitäten wie Wandern, Fitness in einem Park oder Garten, Gartenarbeit, Angeln, Klettern, Joggen oder Radfahren umfassen. Diese körperlichen Aktivitäten können Teil einer täglichen Aktivität sein oder mit einer solchen zusammenfallen, oder es kann sich um einen geplanten Übungszeitraum handeln. Sie können in städtischen, ländlichen oder Wildnisgebieten stattfinden.

4) Bitte bewerten Sie, wie häufig (wie oft) Sie sich in naturreicher Umgebung körperlich betätigen:

- Täglich
- 3 Mal pro Woche
- Einmal pro Woche
- einmal alle zwei Wochen
- Nicht seit drei Wochen

5) Wie viel Aufmerksamkeit widmen Sie der Natur, während Sie sich körperlich betätigen?

|  | Nicht sehr viel  1 | 2 | Ziemlich viel  3 | 4 | Viel  5 |
| --- | --- | --- | --- | --- | --- |
|  |  |  |  |  |  |
