# Supplementary material for: The Importance of Nature Exposure and Physical Activity for Psychological Health and Stress Perception: Evidence From the First Lockdown Period During the Coronavirus Pandemic 2020 in France and Germany
Source: Front Psychol. 2021 Mar 4;12:623946. doi: 10.3389/fpsyg.2021.623946 (PMC7969516; doi:10.3389/fpsyg.2021.623946)
Supplement: Supplementary file 3 [file Table_3.docx]

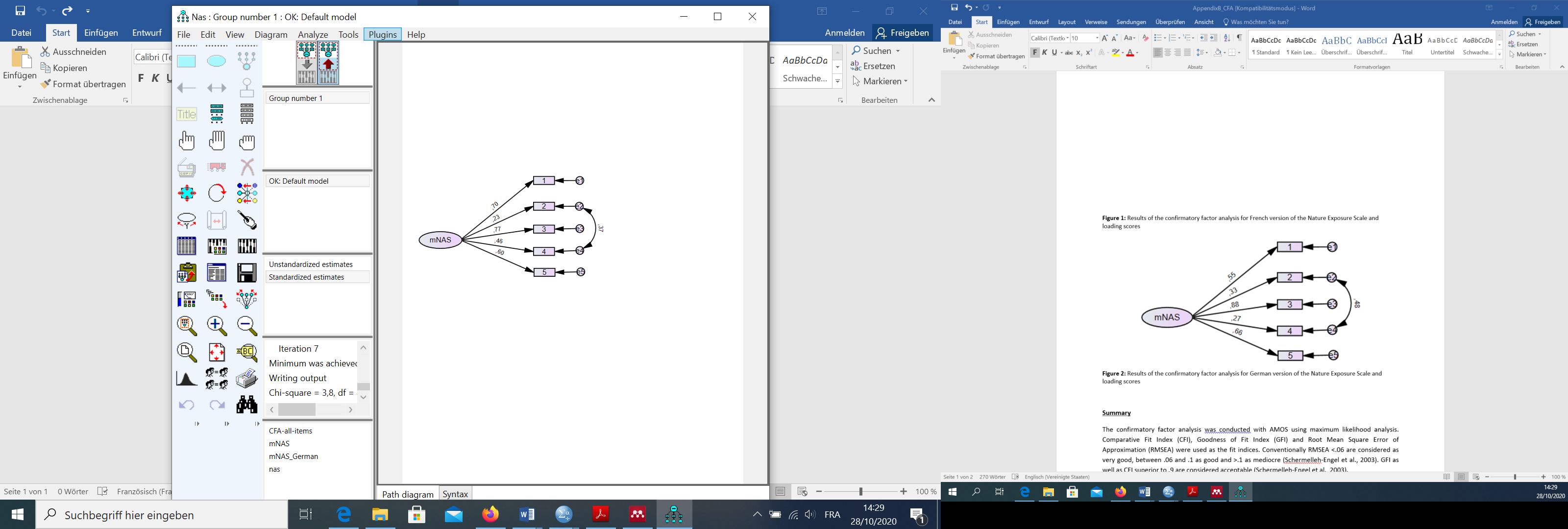


Figure 1: Results of the confirmatory factor analysis for French version of the Nature Exposure Scale and loading scores


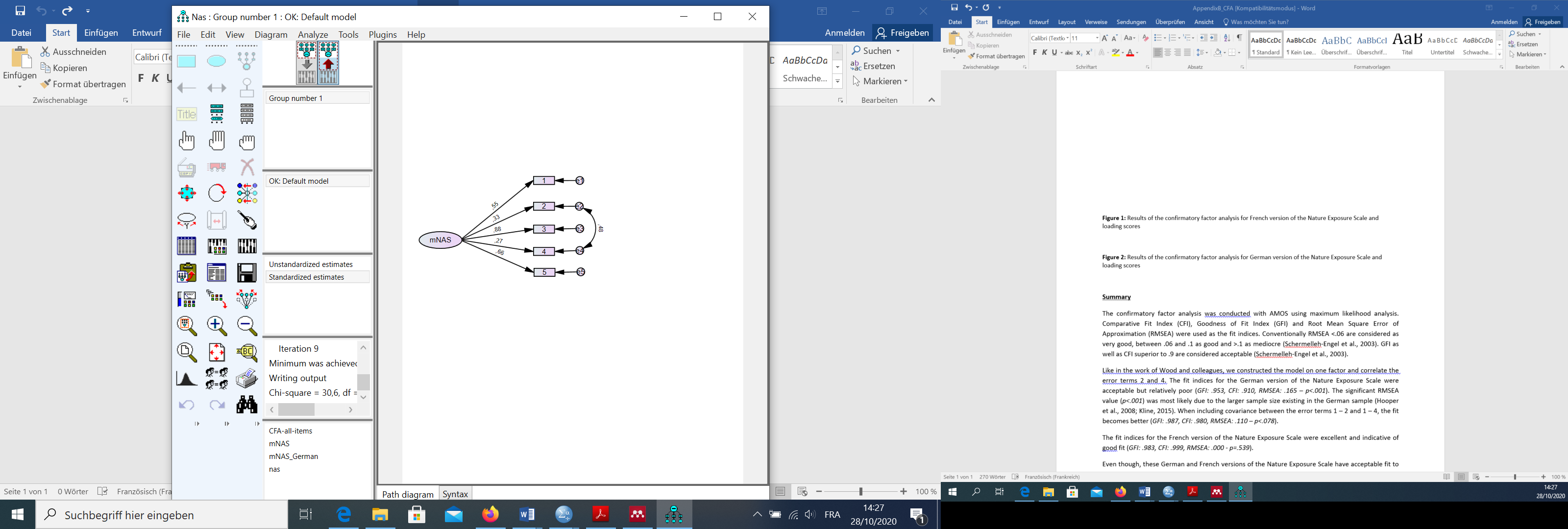


Figure 2: Results of the confirmatory factor analysis for German version of the Nature Exposure Scale and loading scores

**Summary**

The confirmatory factor analysis was conducted with AMOS using maximum likelihood analysis. Comparative Fit Index (CFI), Goodness of Fit Index (GFI) and Root Mean Square Error of Approximation (RMSEA) were used as the fit indices. Conventionally RMSEA <.06 are considered as very good, between .06 and .1 as good and >.1 as mediocre (Schermelleh-Engel et al., 2003). GFI as well as CFI superior to .9 are considered acceptable (Schermelleh-Engel et al., 2003).

Like in the work of Wood and colleagues, we constructed the model on one factor and correlate the error terms 2 and 4. The fit indices for the German version of the Nature Exposure Scale were acceptable but relatively poor (*GFI: .953, CFI: .910, RMSEA: .165 – p<.001*). The significant RMSEA value (*p<.001*) was most likely due to the larger sample size existing in the German sample (Hooper et al., 2008; Kline, 2015). When including covariance between the error terms 1 – 2 and 1 – 4, the fit became better (*GFI: .987, CFI: .980, RMSEA: .110 – p<.078*). The fit indices for the French version of the Nature Exposure Scale were excellent and indicative of good fit (*GFI: .983, CFI: .999, RMSEA: .000 - p=.539*).

Even though, these German and French versions of the Nature Exposure Scale have acceptable fit to be used in our survey, they could be improved and should be re-tested with participants free to go anywhere at will.
